# Dataset of lecturer performance appraisel

**DOI:** 10.1016/j.dib.2020.106161

**Published:** 2020-08-08

**Authors:** Sukirno Sukirno

**Affiliations:** a Accounting Education Department, Faculty of Economics, Yogyakarta State University, Indonesia

**Keywords:** Lecturer performance, Reward, Satisfaction, Commitment, Higher education

## Abstract

The dataset showed in this manuscript belongs to the investigation of determinant of lecturer performance in Indonesia. Semi-closed questionnaires were administered to collect data and 750 questionnaires were distributed by using snowball-sampling method to lecturers, peers, and students in the public and private universities in Indonesia. About 347 questionnaires were returned and could be further analyzed. Respondents were required to indicate their level of agreement on various items with a five-point scale. Exploratory factor analysis was used to test the item validity and Cronbach's Alpha test was employed to examine the instrument reliability. Besides, path analysis was also employed to test research hypotheses. Lecturer performance is an endless issue in education and the data can be used to explore the lecturer performance. Besides, it may also be used in developing an appraisal model of teacher performance other education levels as well.

**Specifications of Data**

**Table:** 

Subject	Education
Specific subject area	Educational Performance Management
Type of data	Table Figure
How data were acquired	Instruments: Semi-closed questionnaires were distributed to lecturers, peers, and students.
Data format	Raw Analyzed
Parameters for data collection	Data were collected by distributing semi-closed questionnaires to lecturers, peers, and students in higher education institution in Indonesia with a snowball sampling technique.
Description of data collection	Data comprise of quantitative and qualitative data.
Data source location	Institution: Higher Education Institions City/Town/Region: Yogyakarta Country: Indonesia The latitude of Yogyakarta City, Yogyakarta, Indonesia is -7.797068, and the longitude is 110.370529. Yogyakarta City, Yogyakarta, Indonesia is located at Indonesia country in the Cities place category with the gps coordinates of 7° 47′ 49.4448′' S and 110° 22′ 13.9044′' E.
Data accessibility	With the article
Related research article	DS Sukirno, S Siengthai Does participative decision making affect lecturer performance in higher education? International Journal of Educational Management https://www.emerald.com/insight/content/doi/10.1108/09513541111146387/full/html

**Value of the Data**

• The data are particularly valuable for researchers that aim to model lecturer performance based on a multirater approach. Beside, the data can also be used to compare an error of measurements between the classical measurement model and the item response theory model (graded response model or partial credit model).
• The data will be useful for researchers to find the fittest model of lecturer performance among different demograpic data.
• The data further may also be added in educational databases that later may benefit relevant parties in conducting further research.

## 1 Data

The sample was drawn by a snowball sampling refering to a data collection procedure by choosing initial respondents randomly, then additional respondents are contacted referring to the information given by the first group of respondents (Zikmund, 1994). A number of 750 units of questionnaires were distributed among lecturers, peers, and students in many universities in Indonesia according to the research design. Each unit of questionnaire comprised of three different questionnaires with different colours and titles. It was prepared to make respondents easier to identify which questionnaire was administered for lecturers, peers, and students. Green colour was designed for the students, pink colour for the lecturers and last yellow colour was designed for the peers.

Data collection was a phase in which the researcher was taking more efforts and time in the field. At the beginning, after being revised, the questionnaires were copied and distributed to the respondents. Following the data collection, coding was carried out for easy entering and analizing of the data. Coding theoretically refers to the set of all tasks associated with ransforming edited responses into a form that is ready for anlysis [21]. The sequence steps of coding process involved:

a categorizing the data based on the similarity.
b assigning numerical codes to the categories.
c creating a data set suitable for computer analysis.

There were two main categories of measurement scale used in this research, nominal and ordinal scale. Detail framework of the data codings are illustrated in Table 1.

**Table 1:** Data Entry

No	Variables	Description	Code	Measure
1.	Demographic Data a. Student	Student’ identities (Three items). (University, Sex, Year Administered).	UNIV_CODE SSEX YEAR	Nominal
	b. Lecturer	Lecturer's identities (twenty items). (Name, University, Faculty, FOS, Sex, Position, Employment Status, Teaching Status, Type of university, Age, Marriage status, Education, Experience, Academic rank, Duration on the Last Rank, Credit Tought, Another business, What field of business, Abstract required, Rater's name).	LA1 – LA20	Nominal
	c. Peer	Peer's identities (twenty items). (Name, University, Faculty, FOS, Sex, Position, Employment Status, Teaching Status, Type of university, Age, Marriage status, Education, Experience, Academic rank, Duration on the Last Rank, Credit Tought, Another business, What field of business, Abstract required, Ratee's name).	SUPA5 – SUPA18	Nominal
2.	Participation in Dec. Making (LECPAR) a. Lecturer	Assessing lecturer's participation (twelve items). Self rating with five points running from 1= never to 5= always.	LP1 – LP12	Ordinal
	b. Peer	Assessing peer's rating on participation (twelve items). Peer rating with five points running from 1 = never to 5 = always.	SUPP1 – SUPP12	Ordinal
3.	Reward System (LECREW) a. Lecturer	Assessing reward system practice (eight items). Self rating with five points running from 1 = never to 5 = always.	LRS1–LRS8	Ordinal
	b. Peer	Assessing reward system practice (eight items). Peer rating with five points running from 1 = never to 5 = always.	SUPRS1–SUPRS8	Ordinal
4.	Lecturer Satisfaction (LECSAT) a. Lecturer	Assessing lecturer's satisfaction (nine items). Self rating with five points running from 1 = strongly dissatisfying to 5 = strongly satisfying.	LS1 – LS9	Ordinal
	b. Peer	Assessing lecturer's satisfaction (nine items). Peer rating with five points running from 1 = never to 5 = always.	SUPS1 – SUPS9	Ordinal
5.	Lecturer Commitment (LECCOM) a. Lecturer	Assessing lecturer's commitment (eighteen items). Self rating with five points running from 1 = never to 5 = always.	LC1 – LC18	Ordinal
	b. Peer	Assessing lecturer's commitment (eighteen items). Peer rating with five points running from 1 = never to 5 = always.	SUPC5 – SUPC18	Ordinal
6.	Lecturer Performance (LECPER) a. Student	Student evaluation on teaching performance (fifteen items). Student rating with five points running from 1 = never to 5 = always	SB1 - SB15	Ordinal
	b. Lecturer	Assessing lecturer's performance (six items). Self rating with five points running from 1 = never to 5 = always.	LPERF1 – LPERF6	Ordinal
	c. Peer	Assessing lecturer's performance (six items). Peer rating with five points running from 1 = never to 5 = always.	SUPPER1 – SUPPER6	Ordinal

A total of 750 packets of semi-closed questionnaires were distributed to lecturers, peers, and students from 39 universities in Indonesia. The choice of using peers and students as raters in this research were initiated from Falchikov and Goldfinch [10], Sanchez et al. [25], and Double et al. [9]. They confirmed that peer assessments tend to be highly correlated with teacher and student assessments. Table 2 below briefly describes the data related to the respondents’ response rates. Per group, respondents from public universities (Lecturer/Peer = 68.80%; Student = 68.40%) had higher number in responding a survey than those who came from private universities (Lecturer/Peer = 50.00%; Student = 59.00%). Furhter, the usable ratio per group was 35.45% (Lecturer/Peer) and 49.20% (Student) for respondents from public universities and 64.55% (Lecturer/Peer) and 44.80% (Student) from its counterpart. In total, response rate of this survey was 56.27% (Lecturer/Peer) and 59.47% (Student). Working closely with the universities for three months, in total, about 347 usable questionnaires (matched between raters) from 39 universities were returned and could be further analyzed which was about 46.27% (Lecturer/Peer/Student) rate of return. The researcher decided to exclude the unmatched questionnaires from the analysis [8]. Hence, because the completed and usable questionnaire response rate was in the amount of 46.27% less than 50%, so the generalizability of this data interpretations might not be appropriate beyond the respondents [7].

**Table 2:** Response Rate

Respondent	University		Questionnaire Distributed	Questionnaire Returned	Usable Questionnaire	Response Rate	Usable Rate
	Type	Number					
1. Lecturer & Peer	Public	6	250	172	123	68.80%	35.45%
	Private	33	500	250	224	50.00%	64.55%
	Total	39	750	422	347	56.27%	46.27%
2. Student	Public	6	250	171	123	68,40%	49.20%
	Private	33	500	295	224	59.00%	44.80%
	Total	39	750	466	347	62.13%	46.27%

## 2 Experimental design, materials, and methods

To collect the data, five semi-closed questionnaires were distributed to the respodents. Firstly, an instrument developed by Marks and Louis (1997) was adopted to measure lecturer performance in this research. It was considered as the most complete instrument in assessing PDM compared to others. Besides, it comprises of all aspects used in the related previous studies. In this case, respondents were required to rate the level of lecturer's participation in decision making indicated by several items using a five-point scale, 1 = never to 5 = always. The first question stated in the instrument administered for the lecturer to indicate his or her own PDM was “How often do you participate in the following aspects of decision making?”. In contrast, the first statement given to peer to rate his or her colleague participation was “Based on my observation, the frequency of my colleague participating in the following aspects of decision making process are: …”. There are three major indicators of PDM consisting of “school operations and management (planning the school building and budget, determining the school schedule, determining specific professional and teaching assignments, establishing the school curriculum, hiring new professional personnel, determining the content of practical subjects), students’ school experiences (determining student behavioral codes, disciplining students, setting policy on grouping students in class by ability) and control over classroom instruction (selecting textbooks and other instructional materials, selecting content, topics and skills to be taught, selecting teaching strategies)”.

Secondly, to assess reward system practices, an instrument developed by Tsai [29] was adopted. The instrument was designed in a five-point Likert scale, 1 = never to 5 = always. There were five aspects of qualified reward system practices were administered. Thirdly, an instrument from Rice and Schneider [22] was administered to measure job satisfaction in education. Respondents were required to indicate their level of agreement on various items with a five-point scale, 1 = strongly dissatisfying to 5 = strongly satisfying. A high rating score indicates a high level of satisfaction and a low rating score indicates a high level of dissatisfaction. Fourthly, this research adopted an eighteen-item of organizational commitment instrument developed by Smeenk et al. [26]. The lecturers and peers were requested to indicate their level of agreement on various statements regarding lecturers or their colleagues’ commitment using a five-point scale, 1 = never to 5 = always. Finally, the lecturer performance instrument was measured by six indicators from Smeenk (2008). The six indicators are relevant to the four elements of the lecturer performance stipulated by Minister of Education and Culture of Republic of Indonesian No. 92/2014 which comprises of teaching, research, publication, and social engagement element performance. Based on the data, the following sections describe an exploratory factor analysis for testing the items validity and a Cronbach's Alpha for testing the instrument reliability for all instruments rated by lecturers, peers, and students.

a Lecturer and Peer Rater

Table 3 provides the validity and reliability analysis of the instruments rated by lecturers and peers. A Kaiser, Meyer, and Olkin (KMO) test was conducted to determine if the items were measuring a common factor as suggested (Robinett, 2008). KMO test for overall variables was 0.908 greater than 0.50 indicating that the instruments rated by lecturers and peers account for a significant amount of variance. Furthermore, the probability associated with the significance values of the Bartlett test of Sphericity was p < 0.000 meaning that the sample inter-correlation matrix totally do not come from a population in which its matrix was identical. Both figures indicated that EFA could be carried out.

**Table 3:** Validity and Reliability Analysis of Instrument for Lecturer and Peer Rater

Variables/Items	Loading Values	
	Lecturer	Peer
Participation in Decision Making (LECPAR)		
1. Planning the school building and budget (LP1)	0.501	0.580
2. Determining the teaching schedule (LP2)	0.655	0.729
3. Determining teaching or other professional assignments (LP3)	0.719	0.772
4. Establishing the curriculum (LP4)	0.683	0.716
5. Selecting new employees (LP5)	0.664	0.694
6. Determining the content of practical subjects (LP6)	0.579	0.599
7. Determining student behavioural codes (LP7)	0.702	0.810
8. Disciplining students (LP8)	0.632	0.682
9. Setting policy on a class size (LP9)	0.582	0.742
10. Selecting textbooks and other instructional materials (LP10)	0.572	0.560
11. Selecting content, topics and skills to be taught (LP11)	0.578	0.539
12. Selecting teaching strategies (LP12)	0.579	0.454
Eigen Value	4.834	11.899
Percentage of Variance	9.121	22.450
Cumulative Percentage of Variance	27.482	22.450
Cronbach Alpa	0.868	0.899
Maximum	0.719	0.810
Minimum	0.501	0.454
Standard Deviation	0.065	0.108
Lecturer satisfaction (LECSAT)		
1. Administration and supervision (LS1)	0.816	0.663
2. Co-workers (LS2)	0.749	0.634
3. Future career (LS3)	0.771	0.707
4. Institutional identification (LS4)	0.761	0.752
5. Financial aspects (LS5)	0.578	0.727
6. Work conditions (LS6)	0.829	0.766
7. Amount of work (LS7)	0.716	0.637
8. Student-lecturer relations (LS8)	0.676	0.585
9. Community relations (LS9)	0.699	0.512
Eigen Value	2.893	2.781
Percentage of Variance	5.458	5.247
Cumulative Percentage of Variance	41.081	49.824
Cronbach Alpa	0.897	0.854
Maximum	0.829	0.766
Minimum	0.578	0.512
Standard Deviation	0.077	0.083
Lecturer commitment (LECCOM)		
1. I hope to be able to spend the rest of my career in this university (LC1)	0.556	0.453
2. I enjoy discussing the negative sides of this university with external people (R) (LC2)	0.440	0.568
3. I feel as if the university's problems are my own (LC3)	0.523	0.668
4. I feel like a part of the family's at this university (LC4)	0.687	0.768
5. This university has a great special meaning for me (LC5)	0.660	0.743
6. I easily become fascinated to another university's facility (R) (LC6)	0.424	0.527
7. I do not care about of what might happen with this university if I quit my present job (R) (LC7)	0.614	0.535
8. It would be very hard for me to leave this university right now (LC8)	0.614	0.323
9. My life would suffer very much if I decided to leave this university (LC9)	0.281	0.038
10. I could leave this university at no cost at any time (R) (LC10)	0.598	0.715
11. I feel that I have too many reasons to leave this university (R) (LC11)	0.648	0.812
12. I continue to work for this university as leaving would require sacrifice (LC12)	0.300	0.235
13. For me, leaving from one to another university very often is unusual (R) (LC13)	0.326	0.242
14. I do not mind at all when employees move from one to another university (R) (LC14)	0.395	0.189
15. If I got offered a job elsewhere I would leave this university (R) (LC15)	0.628	0.645
16. I believe that loyalty is very important to an university (LC16)	0.576	0.514
17. For me, to be an entrepreneur is better than as a lecturer (R) (LC17)	0.406	0.531
18. Things about this university are better since I joined with this university (LC18)	0.193	0.133
Eigen Value	9.731	4.310
Percentage of Variance	18.361	8.133
Cumulative Percentage of Variance	18.361	30.583
Cronbach Alpa	0.846	0.870
Maximum	0.687	0.812
Minimum	0.193	0.038
Standard Deviation	0.150	0.235
Reward System (LECREW)		
1. There is a strong link between how well I perform my job and receive recognition and praise (LRS1)	0.758	0.790
2. There is a strong link between how well I perform my job and receive performance appraisal (LRS2)	0.652	0.772
3. There is a strong link between how well I perform my job and receive an increase in pay/salary (including allowance) (LRS3)	0.788	0.721
4. There is a strong link between how well I perform my job and receive promotion (LRS4)	0.709	0.707
5. University recognition is based on the employees’ performance (LRS5)	0.750	0.711
6. Compensation increases are based on group performance rather than personal performance (LRS6)	0.518	0.656
7. University rewards employees who make an extra effort (LRS7)	0.744	0.777
8. The organization's reward and incentive compensation scheme / package strongly emphasizes employees’ performance (LRS8)	0.746	0.769
Eigen Value	4.315	4.193
Percentage of Variance	8.141	7.912
Cumulative Percentage of Variance	35.624	38.495
Cronbach Alpa	0.902	0.917
Maximum	0.788	0.790
Minimum	0.518	0.656
Standard Deviation	0.087	0.046
Lecturer performance (LECPER)		
1. Teaching performance (LPERF1)	0.613	0.682
2. Research performance (LPERF2)	0.804	0.785
3. Publication performance (LPERF3)	0.769	0.801
4. Public engagement performance (LPERF4)	0.676	0.770
5. Miscellaneous (LPERF5)	0.757	0.722
6. Overall performance (LPERF6)	0.787	0.809
Eigen Value	2.726	3.224
Percentage of Variance	5.144	6.082
Cumulative Percentage of Variance	46.226	44.577
Cronbach Alpa	0.853	0.914
Maximum	0.804	0.809
Minimum	0.613	0.682
Standard Deviation	0.074	0.050
Kaiser-Meyer-Olkin Measure of Sampling Adequacy	0.841	0.858
Bartlett's Test of Sphericity		
Approx. Chi-Square	9,546.768	11,861.956
Degree of freedom	1.378	1.378
Significance	0.000	0.000

Five factors related to the lecturer participation, lecturer commitment, lecturer satisfaction, lecturer performance and reward system practices were investigated. Five interpretations were drawn based on the factor analysis. Firstly, it was found that all participation items (lecturer and peer rating) loaded accordingly in the same factor with loading values greater than 0.50 [13] indicated that the instruments were unidimensional and valid. Latent roots (eigen value) equal to 4.834 (lecturer rating) and 11.899 (peer rating) greater than 1 as it is required (Wise, 1998). Besides, 9.121% (lecturer) and 22.450% (peer) variances of all latent variables was explained by lecturer participation variable. Cronbach's Alpha coefficients shown 0.868 (lecturer rating) and 0.899 (peer rating) indicated that the instruments used to measure lecturer participation were reliable [[13],[20]].

The highest participation in decision making was given by lecturers in determining teaching or other professional assignments (0.719 by lecturer rating) and determining student behavioural codes (0.810 by lecturer rating). Those items indicate that knowledge and skills related to professional development and determining student behavioral codes are the issues that lecturers are the most interested in. Lecturers give the lowest attention on item related to the planning the school building and budget activities (0.501 by lecturer rating) and selecting teaching strategies (0.454 by peer rating).

Secondly, there were nine items with five point scales were administered and distributed to measure lecturer satisfaction [22]. Based on statistical figures listed in Table 4, loading values of all indicators of lecturer satisfaction span from 0.512 to 0.829 loaded in one factor indicated that the instruments were unidimensional and valid (Wise, 1998; [13]). About 5.458% (lecturer rating) and 5.247% (peer rating) of variances could be explained by lecturer satisfaction. Total of eigen value was 4.315 greater than 1 and Cronbach's Alpha coefficients were 0.897 (lecturer rating) and 0.766 (peer rating) again could indicate that the instruments were qualified [[13],[20]]. Loading values for work conditions based on the lecturer as well as peer rating subsequently provide the highest contribution at 0.829 and 0.766. In contrast to the work condition, financial aspects (lecturer rating) and community relations (peer rating) both show the lowest loading values (0.578 and 0.512).

Thirdly, to measure the level of lecturer commitment in educational institution, an instrument developed by Smeenk et al. [26] was adopted. Based on the exploratory factor analysis presented in Table 4, it indicates that from eightteen items administered, the number of items indicate invalid with loading values less than 0.50 were subsequently eight items for gained from the lecturer rating (item number 2, 6, 9, 12, 13, 14, 17, and 18) and seven items from the peer rating (item number 1, 8, 9, 12, 13, 14, and 18).

For the next analysis those eight items from the instrument administered for lecturers and seven items from the instrument administered for peers were excluded. Factors that might affect the problems were respondents’ fatigue and laziness (Ackerman and Ruth, 2009). By entering the remaining items, eigen values for each instrument were subsequently 9.731 (lecturer rating) and 4.310 (peer rating) higher than it was required (Wise, 1998) and Cronbach's Alpha coefficients were 0.846 (lecturer rating) and 0.870 (peer rating). With coefficient alpha greater than 0.70, it can be stated that the instruments used to measure lecturer commitment were reliable [[13], [20]]. In addition, variances total explained from the composite factor were 18.361% (lecturer rating) and 30.583% (peer rating).

The best item representing lecturer commitment is the item stating “I feel like a part of the family's at this university” (lecturer rating) and “I feel that I have too many reasons to leave this university” (peer rating). Those statements explain about 68.70% (lecturer rating) and 81.20% (peer rating) of variances of the lecturer commitment. In contrast, item stating “Things about this university are better since I joined with this university” explains only 19.30% (lecturer rating) and “My life would suffer very much if I decided to leave this university” explains only 3.80% (peer rating) of the variances total of the lecturer commitment. The percentages indicate that lecturers tend to less care of their contribution to the organizational performance and as an indicator of less loyal commitment as well.

Fourthly, eight items indicating reward system practices from Tsai [29] were adopted in this research. It covered both financial and non-financial rewards. Based on the EFA in Table 4, loading values of all items were greater than 0.50 indicated that the items were valid in indicating reward system. Moreover, eigen value was 2.893 (lecturer rating) and 4.193 (peer rating) higher than suggested value (Wise, 1998). Variances explained by the factors were 8.141% (lecturer rating) and 7.912% (peer rating). Lastly, Cronbach's Alpha coefficients were 0.912 (lecturer rating) and 0.917 (peer rating) which are higher than 0.700 indicating that the instruments were reliable.

Table 4 shows that almost all indicators of reward system practices have more than 0.500 loading value. The lowest loading value (0.518 by lecturer rating and 0.656 by peer rating) goes to the item mentioning “Compensation increases are based on group performance rather than personal performance”. It represents that lecturer's rewards were administered based on the personal performance rather than the group performance. Based on the loading scores, the item stating “There is a strong link between how well I perform my job and receive an increase in pay/salary (including allowance)” (0.788 by lecturer rating) and “There is a strong link between how well I perform my job and receive recognition and praise” (0.790 by peer rating) are the best indicator of the reward system practices.

Finally, the exploratory factor analysis on the items of the lecturer performance adopted from Smeenk (2008) has performed well in assessing the lecturer performance. It was indicated by all six items loaded at more than 0.50 nesting in one factor. The composite variable was able to explain 5.114% (lecturer rating) and 6.082 (peer rating) variances. The loadings based on the rating both lecturers and peers range from 0.613 (the lowest) to 0.809 (the highest). The research performance is the most represents lecturer performance in this research (0.804).

Table 4 also shows that KMO for overall variables were subsequently 0.841 (lecturer rating) and 0.858 (peer rating) greater than 0.50 and the probabilities associated with the Bartlett test of Sphericity for this research was p < 0.000 which both are less than the level of significance (0.05). Both indicators indicate no constraint in implementing the exploratory factor analysis [13].

a Student Rater

Table 4 depicts EFA procedures taken to examine the unidemensionality of items used to measure teaching performance rated by students. An instrument with fifteen items was initially developed by Finelli et al. [11] was adopted in this research. After running three phases of a factor analysis and examining anti-image correlation coefficients, three items with the lowest coefficients were excluded. The loading values of all the three phases of factor analysis are sorted in the following table.

**Table 4:** Loading Factor for Teaching Performance

Item	Run I	Run II	Run III
1. I learned a great deal from the lecturer	0.933	0.926	0.931
2. I had strong desire to take this course delivered by the lecturer	0.916	0.911	0.897
3. The lecturer taught in a certain manner to serve students	0.874	0.866	X
4. The lecturer gave clear explanations	0.927	0.935	0.931
5. The lecturer was enthusiastic	0.919	0.913	0.920
6. The lecturer responded all students’ questions	0.939	0.933	0.933
7. The lecturer treated students with respect	0.903	0.932	0.947
8. The lecturer was willing to meet and help students outside of class	0.870	X	X
9. The lecturer kept students informed of their progress	0.928	0.929	0.922
10. The lecturer used class time well	0.913	0.900	0.896
11. The lecturer seemed well prepared for each class	0.927	0.934	0.928
12. Work requirements and grading system were clear from the beginning	0.905	0.947	0.938
13. The amount of work required was appropriate for the credit received	0.925	0.913	0.891
14. The lecturer set high standards for students	0.828	X	X
15. The lecturer used a certain technique that motivated students’ participation in the class	0.883	0.872	0.910
Number of Loading Factor	3	2	1
Eigen Value 1	6.476	6.009	5.623
Eigen Value 2	1.326	1.091	-
Eigen Value 3	1.020	-	-

X = Item is excluded from EFA

In the third phase, all items have loaded into one dimension with latent roots (eigen value) of 5.623 which is greater than one as it is required (Hair et al., 1992). Based on the loading values, three following items were deleted:

1) The lecturer taught in a certain manner to serve students.
2) The lecturer was willing to meet and help students outside of class.
3) The lecturer set high standards for students.

Based on the EFA as it is presented in Table 5 below, all items nest properly in one dimension with having more than 0.50 loading values [13]. Measure of sampling adequacy (MSA) measured by the Kaiser-Meyer-Olkin (KMO) statistics shows that KMO for overall variables were 0.921 greater than 0.50, indicating that EFA could be continued. Besides, the probability associated with the Bartlett test of Sphericity for this research was p < 0.000 less than the level of significance (0.05) as it was required. By incorporating twelve items with loading value more than 0.50, Cronbach's Alpha coefficient for the teaching performance instrument was 0.893 higher than it is required 0.70 [[13], [20]]. Data interpretations show that the highest student's rating (4.363) was given on the item saying “The lecturer responded all students’ questions” and the lowest (3.510) was given on the item saying “The lecturer kept students informed of their progress”. On the average, lecturers’ teaching performance was perceived “good” by students.

**Table 5:** Factor Analysis and Reliability Analysis of Instrument for Student Rater

Items	Mean	Std. Deviation	Loading
1. I learned a great deal from the lecturer	3.790	0.952	0.723
2. I had strong desire to take this course delivered by the lecturer	3.692	1.094	0.552
3. The lecturer gave clear explanations	3.974	0.885	0.736
4. The lecturer was enthusiastic	4.075	0.900	0.782
5. The lecturer responded all students’ questions	4.363	0.801	0.655
6. The lecturer treated students with respect	4.288	0.842	0.678
7. The lecturer kept students informed of their progress	3.510	1.004	0.663
8. The lecturer used class time well	3.925	0.968	0.757
9. The lecturer seemed well prepared for each class	3.902	0.957	0.771
10. Work requirements and grading system were clear from the beginning	4.127	0.989	0.637
11. The amount of work required was appropriate for the credit received	3.755	0.931	0.588
12. The lecturer used a certain technique that motivated students’ participation in the class	3.870	0.908	0.630
Eigen Value			5.623
Percentage of Variance			46.859
Cumulative Percentage of Variance			46.859
Cronbach's Alpha			0.893
Kaiser-Meyer-Olkin Measure of Sampling Adequacy.			0.921
Bartlett's Test of Sphericity Approx. Chi-Square Degree of freedom Significance			1,698.134 66 0.000

Loading values listed in Table 5 infer that lecturers’ enthusiasm in teaching has the higest contribution to their teaching performance (0.782). Nevertheless, courses delivered by the lecturers indicate the lowest teaching performance among lecturers (0.552). Based on the validity and reliability test, latent variables then were composed. Only items having more than 0.50 loading values were included in formulating latent variables. By using a formula developed by Sekaran (1992), all variables could be reformulated as follows.

1) Lecturer Rating on Participation in Decision Making (PDM), Lecturer Satisfaction, Lecturer Commitment, Reward System and Lecturer Performance.
  LECPAR=(LP1+LP2+LP3+LP4+LP5+LP6+LP7+LP8+LP9+LP10+LP11+LP12)/12
  LECSAT=(LS1+LS2+LS3+LS4+LS5+LS6+LS7+LS8+LS9)/9
  LECCOM=(LC1+LC3+LC4+LC5+LC7+LC8+LC10+LC11+LC15+LC16)/10
  LECREW=(LRS1+LRS2+LRS3+LRS4+LRS5+LRS6+LRS7+LRS8)/8
  LECPER=(LPERF1+LPERF2+LPERF3+LPERF4+LPERF5+LPERF6)/6
2) Peer Rating on Participation in Decision Making (PDM), Lecturer Satisfaction, Lecturer Commitment, Reward System and Lecturer Performance.
  $$\begin{array}{c} \text{PEERPAR}=(\text{SUPP}1+\text{SUPP}2+\text{SUPP}3+\text{SUPP}4+\text{SUPP}5+\text{SUPP}6+\text{SUPP}7+\text{SUPP}8+\text{SUPP}9+\\ \text{SUPP}10+\text{SUPP}11)/11 \end{array}$$
  PEERSAT=(SUPS1+SUPS2+SUPS3+SUPS4+SUPS5+SUPS6+SUPS7+SUPS8+SUPS9)/9
  $$\begin{array}{c} \text{PEERCOM}=(\text{SUPC}2+\text{SUPC}3+\text{SUPC}4+\text{SUPC}5+\text{SUPC}6+\text{SUPC}7+\text{SUPC}10+\text{SUPC}11+\text{SUPC}15\\ +\text{SUPC}16+\text{SUPC}17)/11 \end{array}$$
  PEERREW=(SUPRS1+SUPRS2+SUPRS3+SUPRS4+SUPRS5+SUPRS6+SUPRS7+SUPRS8)/8
  PEERPER=(SUPPER1+SUPPER2+SUPPER3+SUPPER4+SUPPER5+SUPPER6)/6
3) Student Rating on Teaching Performance
  STUPER=(SB1+SB2+SB4+SB5+SB6+SB7+SB9+SB10+SB11+SB12+SB13+SB15)/12.

Based on the data analysis, mean values of respondents’ perception on lecturer performance, reward system practice, PDM, lecturer satisfaction, and lecturer commitment span from around 3 (moderate level) to 4 (high level). Lecturers perceived their own participation in decision making (LECPAR) in the moderate level (3.121). Accordingly, peer's perception their colleagues in PDM was also almost in the same level (3.152). The similarity of perception on PDM was again observed in other variables respectively between lecturer and peer on reward system practices (3.229 and 3.316), satisfaction (3.614 and 3.609) and performance (3.633 and 3.657), except perception on commitment. Lecturers rated their own perception (4.096) higher compared to their peers rating (3.794). In term of the lecturer performance, students perceived better on lecturers’ teaching performance (3.939) which is higher than the perception given by lecturers (3.633) and peers (3.657).

Table 6 shows that means matrix of the lecturer performance constructs are different among raters. On average, the teaching performance construct was perceived in the highest level subsequently followed by the social engagement and research construct, while the publication construct was perceived in the lowest level at 3.419. The gradation has confirmed that the bigest factor hindering the lecturer performance among Indonesian lecturers was on publication [2].

**Tabel 6:** Mean Comparison of Lecturer Performance Constructs

Performance Element	Rater			Average
	Lecturer	Peer	Student	
Teaching	3.865	3.919	3.939	3.908
Research	3.513	3.548	-	3.530
Publication	3.418	3.421	-	3.419
Social Engagement	3.635	3.723	-	3.679

Several sections below provide evidence related to the relationship between factors affecting lecturer performance and lecturer demographic characteristics refining a part of the previous research objectives done by Fincham and Rhodes (1994), Bogler (2002) and Bull [4]. Bull [4] and Chughtai [6] revealed that gender, tenure, age, educational level and job level have a positive and significant relationship with lecturer satisfaction and lecturer commitment.

**Fig. 1:**
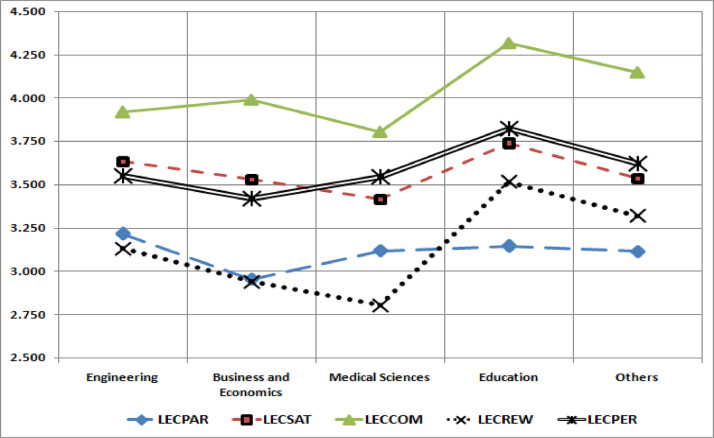
Mean Comparison of Lecturer Rating Based on School Backgrounds

Fig. 1 shows that the level of participation, performance, and rewards of lecturers working in the school of business and economics were in the lowest position. The higher number of students and sidejobs of lecturers in the business and economics school compared to other schools usually becomes the first factor influencing the condition. Lecturers in the school of business and economics in Indonesia have to teach more credits and do more sidejobs has affected their low performance compared to other lecturers from other school backgounds. In contrast, medical schools lecturers perceived their satisfaction and reward in the lowest level.

**Fig. 2:**
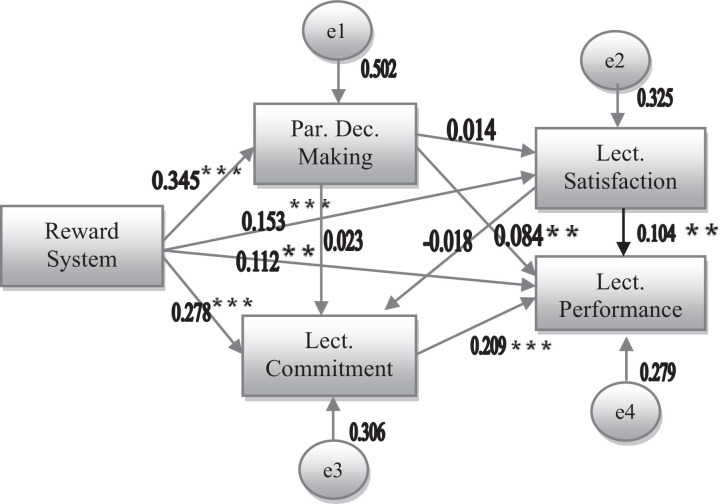
Original Model on Lecturer Performance *χ^2^* *=* *187.108; p*  *=* *0.000; GFI*  *=*  *0.793; AGFI*  *=*  *0.690; RMSEA*  *=*  *0.226* *= p  *<*  0.10; **= p  *<*  0.050; ***= p  *<*  *0.001*

After describing the statistics of variables and validating the instruments, the next section is describing the path analysis. First run of the path analysis of the model was not fit, since the fitness indices did not meet the requirements. Chi-Square was still more than 3, GFI and AGFI were less than 0.9 [13] and RMSEA was 0.226 which is more than acceptable rate [15]. Graphical presentation of the relationship among variables affecting lecturer performance is presented in the following Fig. 2.

**Figure 3:**
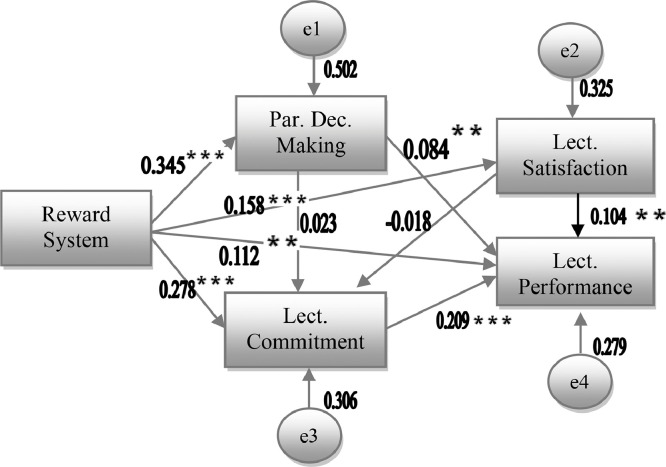
Revised Model based on Lecturer Performance χ^2^  =  0.104; p  =  0.747; GFI  =  1.000; AGFI  =  0.998; RMSEA  =  0.000 *= p  *<*  0.10; **= p  *<*  0.050; ***= p  *<*  *0.001*

Consequently, the model was respecified by performing a competing model strategy [13]. In order to obtain more parsiomonious and clearer model, non-significant effects (p > 0.05) were excluded from the initial model [31]. By deleting the path arrow representing the relationship between PDM and the lecturer satisfaction, the empirical model became fitter. The following figure is the revised model of the path analysis.

Based on the path analysis, direct and indirect effect as well as total effect can be identified. The total effect of one variable (reward system) on another (lectuer performance) is the sum of the indirect (0.345 + 0.084 via PDM; 0.278 + 0.209 via lecturer commitment; 0.158 + 0.104 via lecturer satisfaction) and the direct effect (0.112) between them [13]. The direct effect is the relationship between two variables with a single row (eq. the direct effect of reward system on lecturer commitment is 0.278), while the indirect effect is those relationship involving the sequence of relationship of two or more direct effects and is represented visually by multiple arrows (eq. the indirect effects of reward system on lecturer performance consist of 0.278 and 0.209). Such interactions causes lecturercommitment potentially functions as an intervening variable [[18], [28]].

There are five indirect effects that could be identified from the model. First, the relationship between reward system and lecturer satisfaction (0.005 = 0.345 × 0.014). Secondly, the relationship between reward system and lecturer commitment (0.005 = ((0.345 × 0.023)+(0.345 × 0.014 x -0.018)+(0.153 x -0.018)). Thirdly, the relationship between lecturer satisfaction and lecturer performance (-0.004 = -0.018 × 0.209). Fourthly, the relationship between lecturer participation and lecturer performance (0.006 = ((0.014 × 0.104)+(0.023 × 0.209)). Finally, the relationship between reward system and lecturer performance (0.104 = ((0.278 × 0.0.209)+(0.345 × 0.084)+(0.345 × 0.014 × 0.104)+(0.153 × 0.104)). Only one indirect effect is existed in the relationship between PDM and lecturer commitment.

## 3 Policy implications

Relating to the previous data interpretations, recommendations are addressed to the education policy makers. Firstly, providing a reward system that links to performance is believed can be used to motivate and improve lecturer performance in HEIs in Indonesia. Beside using a teacher portfolios (teaching performance, research, publication, public engagement, and managerial involvement), it is also suggested that reward system should be based on the group performance and student performance, and classroom observations.

Secondly, it is very urgent for education policymakers and leaders to keep concern on providing a better supportive administration and supervision system, peers, career in the future, university identification, financial supports, and work conditions for the lecturers. Thirdly, loyal lecturers would feel enjoyable to stay in an organization and strongly believe in organizational values and they would perform better for their organizations. In this case, reward system still could be used to promote lecturer commitment and performance. By considering the condition of school bacgrounds and increasing the lecturer participation in in finding the best reward system and performance evaluation model, policy makers will be able to fit between lecturer reward, satisfaction, and performance. Lecturers must see the rewards as attainable in order for them to engage in the necessary effort to obtain them. Lecturer performance will be better managed when each indicator of lecturer performance is strongly linked with each indicator of reward system, lecturer satisfaction, and lecturer commitment. Finally, its strong relation between factors certainly will boost lecturer and university performance.

It is very important to describe the limitations of the present research. Subsequently, four concerns were identified in which they might decrease the power of generalizability of the data interpretations. Firstly, all measurements consist only of self-assessment items which ask respondents to provide ratings of lecturer performance and its determinants [14]. Over or under-estimates were more likely to be found if the self-assessments are employed in a survey. Young people may over-estimate because they lack the cognitive skills to integrate information about their abilities and were more vulnerable to wishful thinking [24]. Self-assessment might also possess bias against a particular sex, social class, nationality, or racial group [1]. To examine influence of the bias issues, more studies are still needed.

Secondly, by using Slovin method, the sample size should be at least 385 people of each group [30]. A total of 750 questionnaires were distributed to each group of rater in 39 different universities in Yogyakarta Province, nevertheless the response rate was lower than expected. Only 347 people per group participated (± 46% rate or return). Because the completed and usable questionnaire response rate was in the amount of 54% only slightly greater than 50%, so the generalizability of this data interpretations might not be appropriate beyond the respondents [7].

Thirdly, the number of variables included in the model and the model itself that had been developed in this research might not be able to fully represent theoretical and empirical expectation. Regarding this issue, Griffiths [12] argued that there is no hope of doing perfect research. Research is like a continuous, never ending jigsaw puzzle [3]. Many elements should be added to the model before we can have the whole description of an object we are investigating. Good research still needs to improve meaning that there are areas in which a research program is excellent, but some other things may be out of its control altogether [16].

To address the research limitations and to conduct more reliable and rigorous research, three recommendations are proposed. First, in addition to self-rating, it was suggested to use random sampling, different triangulation methods such as gathering data through different time frames, broadening sampling area (different provinces or islands or countries), different format of data (secondary data), or involving a variety of raters (supervisor or head of department).

Second, to increase the generalizability of the data interpretations, sample size and response rate should be increased. Five strategies to increase response rate are developing clear instructions, purpose and questions, motivating the respondents to respond, making respondents interested to the survey, providing reasonable time and ease of completing the survey, and providing incentives and rewards for completion [27]

Finally, another suggestion is related to the variable being investigated. To provide more complete picture of factors affecting lecturer performance, it is recommended to consider other variables such as ability, motivation, effort, selection practices, training and development, employee relations and organization strategy [19] and organizational culture, organizational structure, job stress and leadership style [[5],[17],[18],[23]].

## Declaration of Competing Interest

The author declares no competing financial interests or personal relationships that could have appeared to influence the work reported in this paper.
